# Arthroscopic shoulder suspensioplasty in painful hemiplegic shoulder subluxation—a case series

**DOI:** 10.1016/j.jseint.2023.11.001

**Published:** 2023-11-29

**Authors:** Olivier Bozon, Vittoria Casamenti, Flavia Coroian, Isabelle Laffont, Bertrand Coulet

**Affiliations:** a Upper Limb Surgery Unit, Department of Orthopaedic Surgery, Hospital Lapeyronie, CHU Montpellier, Montpellier, France; b Physical Medicine and Rehabilitation Department, Hospital Lapeyronie, Montpellier, France; c Institut de Neuro-Orthopédie Montpellier INOM, Hospital Lapeyronie, Montpellier, France

**Keywords:** Shoulder subluxation, Hemiplegic, Stroke, Arthroscopy, Suspensioplasty, Pain

## Abstract

**Background:**

Inferior glenohumeral subluxation (GHS) can cause disabling pain in hemiplegics. Conservative treatments have not been proven to be effective or maintained over time. A few studies have shown the benefits of surgical treatment. The objective of our study was to evaluate the medium-term clinical and radiological results of arthroscopic glenohumeral suspensioplasty surgery by biceps tenodesis in the setting of painful GHS in hemiplegics.

**Methods:**

We conducted a retrospective study of patients who underwent arthroscopic glenohumeral suspensioplasty. The assessment, at a minimum of 1 year, included a clinical evaluation (pectoralis major spasticity, pain, range of motion, satisfaction) and a radiographic evaluation (Dursun classification, height of subacromial space).

**Results:**

Five patients with a mean age of 51 years (36-72 years) were included at a mean follow-up of 40 months (12-70). Satisfaction was good in 80% of patients. Pain decreased in all patients, but not significantly. Four patients (80%) would repeat the procedure if it were necessary. In all patients, a reduction in GHS over time was observed, with a reduction in subacromial height, except in 1 patient who suffered a tenodesis rupture during a fall.

**Conclusion:**

Our results suggest that arthroscopic glenohumeral suspensioplasty by biceps tenodesis may be a therapeutic option in hemiplegic patients with painful GHS.

Hemiplegic shoulder pain (HSP) is a frequent complication after stroke, with an incidence of between 22% and 54%.^7^^,^^11^^,^^14^^,^^18^ It leads to difficulties in rehabilitation, alterations in quality of life and increased rates of depression.^14^^,^^18^ Risk factors leading to HSP have been identified, such as the presence of associated sensory or motor deficits,^7^ spasticity of the shoulder muscles,^18^ injury to the supraspinatus tendon, tendinitis of the long head of the biceps (LHB), or more rarely, inferior glenohumeral subluxation (GHS).^11^ Glenohumeral subluxation (GHS) is defined as an abnormal gap between the inferior surface of the acromion and the superior surface of the humerus. During the recovery phase after a stroke, the loss of muscle tone, particularly of the supraspinatus and deltoid, leads to inferior subluxation of the humeral head and tension on the inferior capsule and glenohumeral ligaments. The contribution of GHS in HSP is subject to debate.^12^^,^^22^ However, some authors contend that this pain can be attributed to tension in the richly innervated lower capsule and ligaments.^4^^,^^6^

Many conservative treatments have been described in the management of painful GHS.^1^ They include splinting in a reduced position,^16^ strapping^10^ or electrical stimulation.^15^ However, no method has been proven to be superior or to maintain its effectiveness over time, especially in chronic GHS.^13^^,^^23^

If pain decreases after the subluxation is reduced by splinting the elbow to the body, confirmed by radiographic assessment, surgical management of painful GHS has been proposed by Pinzur and Hopkins,^19^ Namdari and Keenan,^17^ and Thomas and Kim.^21^ More recently, an arthroscopic glenohumeral suspensioplasty surgery by biceps tenodesis was proposed.^3^

The objective of our study was to evaluate the midterm clinical and radiological results of arthroscopic glenohumeral suspensioplasty surgery using biceps tenodesis in the setting of painful GHS in hemiplegics.

## Materials and methods

### Patients

We conducted a single-center retrospective study of adult patients with hemiplegia of vascular origin who underwent arthroscopic glenohumeral suspensioplasty via biceps tenodesis for painful GHS between April 2014 and April 2021 and who had a minimum 1-year follow-up. All patients underwent a preoperative trial with an elbow brace to increase shoulder coaptation. If their pain decreased, this led to an indication for suspensioplasty.

All patients were operated on by the same senior surgeon (BC) specialized in functional surgery of the upper limb. Patients operated using a surgical technique other than the one described, patients lost to follow-up, minor patients, and patients with insufficient follow-up were excluded from the study.

The mean follow-up time was 40 months (12-70). The mean time from onset of paralysis to surgery was 49 months (20-73). All patients were evaluated clinically by an independent examiner at the last follow-up.

The study was approved by our institutional review board (IRB-MTP_2022_01_202101005), and written consent was obtained from all patients. The authors followed the Strengthening the Reporting of Observational Studies in Epidemiology guidelines in writing the study.

### Surgical technique

**Figure 1:**
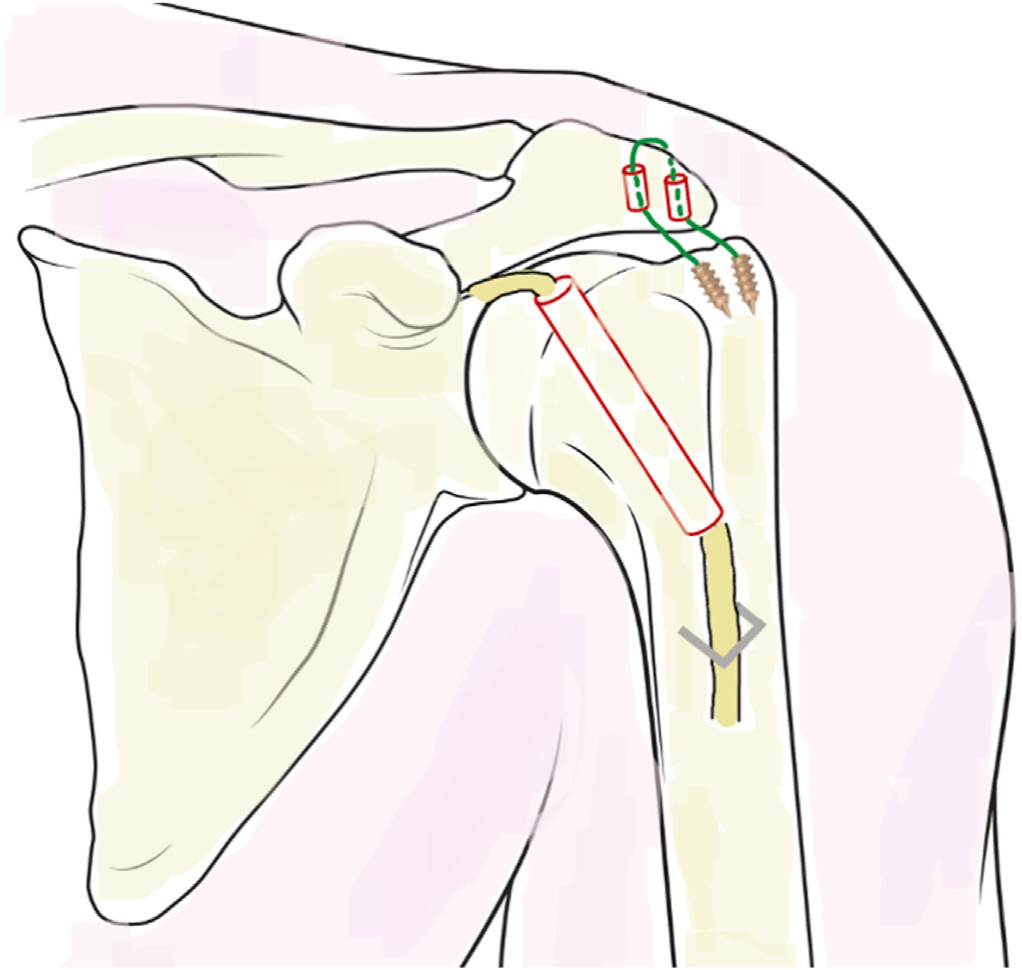
Drawing of the glenohumeral suspensioplasty procedure using biceps tenodesis, according to Bozon and Coulet.^3^

The procedure performed was that proposed by Bozon and Coulet^3^ (Fig. 1). Patients were placed in the beach chair position under general anesthesia. The LHB was cut distally by a short deltopectoral approach, as it exits the bicipital groove. A tunnel was made arthroscopically from the anterior cortex of the humeral shaft to the top of the humeral head. The LHB was pulled into the joint and passed proximally to distally through the humeral tunnel. Two anchors were inserted into the greater tuberosity and their suture tapes passed through two tunnels made through the acromion. The strands formed a loop over the apex of the acromion to enable the humerus to be suspended from the acromion after reducing manually the inferior subluxation. The biceps tendon was then fixed to the humeral shaft using a Blount staple. The tenodesis was protected by a splint for 6 weeks without any mobilization. After this period, gradual removal of the splint was permitted.

### Clinical assessment

Demographic characteristics collected included age, sex, type of first motor neuron damage, and side of the affected hemisphere and dominant side. We documented previous treatment of spasticity in the paralyzed upper limb with botulinum toxin injections, and surgical or traumatic history of the operated shoulder.

Preoperatively, a multidisciplinary evaluation was performed by a physical medicine and rehabilitation physician (IL) and an orthopedic surgeon (BC). A postoperative evaluation at the last follow-up was performed by two independent evaluators—a physical medicine and rehabilitation physician (VC) and an orthopedic surgeon (OB). This evaluation included a functional assessment of proximal and distal control using the House^9^ and Enjalbert^5^ methods, an analytical determination of discriminative and proprioceptive tactile sensitivities, measurement in degrees of passive joint amplitudes in forward elevation, abduction, external and internal rotation. The presence of muscle spasticity in the pectoralis major was also assessed according to the modified Ashworth scale.^2^ Preoperative and postoperative pain in the affected shoulder was quantified with a visual analog scale.

Two simple questions were asked at the last follow-up and included a measure of overall satisfaction with the procedure by a Likert scale (very dissatisfied, somewhat dissatisfied, satisfied, very satisfied), and whether the patient would undergo the procedure again if necessary. Any complications and their nature were noted.

### Radiological assessment

A radiographic workup of the shoulder without splinting (AP and lateral views) was performed. The AP radiographs were analyzed to classify the inferior GHS as described by Dursun (Table I) [8] and to quantify the subacromial height in millimeters.

**Table I:** Dursun classification of inferior glenohumeral subluxations.^4^

Degree of subluxation	Definition
0	Normal: the whole curvature of the glenoid fossa is opposed by and parallel to the humeral head
1	V-shaped widening: the whole curvature of the glenoid fossa is opposed by the humeral head, but V-shaped widening is present in the superior intra-articular space
2	Moderate subluxation: inferior subluxation of the humeral head, with the most superior margin of the humeral head not below the line perpendicularly bisecting the line connecting the most superior and inferior margins of the glenoid fossa
3	Advanced subluxation: similar to moderate subluxation but the superior margin of the humeral head is level with or below the bisecting line
4	Dislocation: the most superior margin of the humeral head is level with or below the most inferior margin of the glenoid fossa.

### Statistical analysis

Data were processed using SPSS software (IBM Corp., Armonk, NY, USA). Normality of the data was assessed using the Shapiro test for quantitative variables. Categorical variables (expressed as numbers and percentages) were compared using the Chi^2^ test or Fisher's exact test depending on whether the conditions for each test were met. Continuous data with a normal distribution were presented as means and standard deviations (SD) or median and ranges otherwise. Preoperative and postoperative comparisons for paired samples were performed using the paired Student *t* test for normally distributed data and the Mann–Whitney test otherwise. The significance level was set at *P* < .05.

## Results

### Patients

Between April 2014 and April 2021, 10 patients underwent glenohumeral suspensioplasty as a treatment of their painful GHS. Five patients were excluded: 3 patients underwent a different suspensioplasty technique and 2 were lost to follow-up. Five patients, 4 women and 1 man, mean age 51 years (36-72 years), were included in the study. The left side was involved in 3 cases (60%) and the dominant side in 3 cases (60%). One patient had suffered a previous surgical neck fracture in the operated shoulder before the paralysis. No shoulder pathology was associated with the inferior GHS on the preoperative imaging workup or during arthroscopic exploration. The causes of the palsy and treatments before the surgery are summarized in Table II. The mean time from onset of paralysis to surgery was 49 months (20-73). The mean follow-up was 40 months (12-70).

**Table II:** Clinical data of the 5 patients.

	Age (y)/sex	Dominant side involved	Cause of the paralysis	Duration of paralysis	Prior treatments	Preoperative pain (VAS)	Postoperative pain (VAS)	Follow-up	Postsurgery satisfaction	Undergo procedure again?
Patient 1	43/woman	No	Hemorrhagic stroke	5 y	Splinting, Botox, electrical stimulation	5	4	6 y	Satisfied	Yes
Patient 2	36/woman	Yes	Hemorrhagic stroke	3 y	Splinting	6	3	6 y	Satisfied	Yes
Patient 3	52/woman	No	Hemorrhagic stroke	2 y	Splinting	8	7	3 y	Somewhat dissatisfied	No
Patient 4	72/man	No	Hemorrhagic stroke	6 y	Splinting, Botox∗	6	4	1 y	Satisfied	Yes
Patient 5	50/woman	Yes	TBI	5 y	Splinting, Botox	6	5	1 y	Satisfied	Yes

*VAS*, visual analog scale; *TBI*, traumatic brain injury.

∗ Botulinum toxin injection.

### Clinical data

In all patients, the involved limb was nonfunctional, with a House score of 0 and a mean Enjalbert stage of 0.4 ± 0.5 (0-1), which was unchanged postoperatively. Analytical determination of sensitivities did not reveal any tactile discriminatory capacity, but found some preserved proprioception in 2 patients, again unchanged postoperatively at the last review. The passive joint range of motion (ROM) is summarized in Table III. There was an overall, but not significant, decrease in mobility.

**Table III:** Preoperative and postoperative passive range of motion (ROM) at last follow-up.

	Preoperative ROM (degrees)	ROM at last follow-up (degrees)	*P* value
Forward elevation	87 ± 27 (45-120)	76 ± 27 (30-100)	.536
Abduction	81 ± 20 (45-90)	65 ± 37 (10-100)	.567
External rotation	30 ± 19 (10-60)	14 ± 15 (0-30)	.237
Internal rotation	50 ± 10 (40-60)	55 ± 16 (40-80)	.567

*ROM*, range of motion.

There was a nonsignificant increase in pectoralis major spasticity at the last follow-up (*P* = .059), from 0.8 ± 0.4 (0-1) to 1.8 ± 0.8 (1-3) according to the modified Ashworth scale. Pain on visual analog scale decreased from 6.2 ± 1.1 (5-8) preoperatively to 4.6 ± 1.5 (3-7) at the last follow-up (*P* = .092). At the last follow-up, 4 patients (80%) were satisfied with the procedure, and 1 patient was somewhat dissatisfied. Four patients (80%) would repeat the procedure if necessary. All patients noted a reduction in pain on examination.

Only 1 complication was noted: a recurrence of painful inferior subluxation following a fall on the shoulder at 3 years postoperative. This recurrence resulted in a rupture of the biceps tenodesis and required open surgical revision using another technique.

### Radiological data

Preoperatively, the mean inferior GHS was 2.2 ± 1 according to the Dursun classification (1 grade 1, 2 grades 2, 2 grades 3) and the mean subacromial height was 20 mm ± 5 (12-25). Postoperatively, the mean inferior GHS was 0.6 ± 1 (0-1) according to Dursun's classification (*P* = .022) and the subacromial height was 12 mm ± 5 (7-19) (*P* = .052). No humeral or acromial fractures or osteolysis was noted at the last follow-up.

## Discussion

Shoulder pain in hemiplegics is multifactorial. Following the poststroke flaccid period, inferior subluxation may be the result of complete atony of the suspensory muscles of the humerus and may be one of the causes of this shoulder pain. We report here the midterm clinical and radiological results of an original technique of arthroscopic suspensioplasty by biceps tenodesis.

Pinzur and Hopkins were the first to propose open biceps tenodesis on the coracoid in a series of 6 hemiplegic patients with painful GHS in 1986.^19^ At a mean follow-up of 2 years, they found complete pain relief in five patients (83%) and no recurrence of the subluxation. Nevertheless, postoperatively, patients were advised to keep the brace on permanently. In 2010, Namdari and Keenan published the results of a humeral suspensioplasty technique using open transosseous biceps fixation in a series of 11 patients.^17^ Postoperatively, the patients’ arms were immobilized for 3 months in a sling. At a mean follow-up of 3 years, pain reduction was noted in all patients, and no patient was using a sling at the last follow-up. Tenotomy of the pectoralis major was also done in 7 patients and a reduction in spasticity was noted at the last follow-up. One complication was reported (hematoma). More recently, Thomas and Kim reported the results of a modified Keenan technique, with the addition of biceps tendon suturing at the rotator interval, in 5 patients at a mean follow-up of 3.2 years.^21^ They found significant pain reduction, without modification of the passive shoulder ROM. Radiologically, persistence of the GHS was found in all patients at the last follow-up.

Our study reports results from a patient population similar to previous studies. However, many results and observations differ. In contrast to Keenan, we found a decrease in passive ROM at the last review, which we attribute to a stiffening but deliberate effect of tenodesis on the glenohumeral joint. The reduction in pain was not as dramatic as reported in previous studies, although it occurred in all patients. We propose several explanations for these differences. First, no tenotomy procedure was performed on the internal rotators of the shoulder such as the pectoralis major, which in Keenan's series had resulted in a significant reduction in pain and spasticity in the subgroup of patients who had undergone tenotomy of the pectoralis major. Preoperatively, in our series, 3 patients (60%) received botulinum toxin injections preoperatively, while none did postoperatively. We believe that the increase in spasticity at the last review in our series may have led to residual pain, since at the last review, all the patients had a favorable change in the type of pain between preoperative and postoperative stages, without being able to quantify it. Pain in hemiplegics is multifactorial and difficult to evaluate. The use of more specific pain scales would probably confirm our clinical impression. Secondly, the retrospective nature of the study may also have contributed to bias in pain assessment in these patients whose upper limb is nonfunctional and a source of significant discomfort. Moreover, the duration of immobilization recommended in our protocol was only 6 weeks, which is less than that recommended in the other studies (8 weeks for Thomas, 3 months for Keenan, and for life for Pinzur). We think that the 6-week immobilization period is probably insufficient and could be a contributor to the residual pain, inducing maintenance of painful stimuli and reflex spasticity.

Regarding the surgical technique, our radiological results attest to the maintenance of GHS reduction over time, except in the patient who fell on her shoulder. The use of anchor suspension with wires passed through the acromion provides optimal primary stability, allowing better biological integration of the biceps over time. The proposed technique and the use of arthroscopy offer the interesting possibility of checking the quality of the biceps tendon tissue, and of being able to repair any associated rotator cuff injuries in the same operation. However, this situation was not found in our case series. The value of arthroscopy in shoulder surgery has been evaluated in numerous studies. Although it has been shown that patients prefer arthroscopic surgery,^20^ its superiority is not always demonstrated.^8^^,^^24^ It might therefore be interesting to compare, in a case-control study, the arthroscopic technique described here with an open surgical technique.

Our study has several limitations. The number of patients reported is small, as found in other series in the literature, reflecting the rarity of these procedures, which are solely performed in specialized centers. However, our study contains a series of homogeneous patients reviewed at an average follow-up of more than 3 years. The retrospective nature of the study may give rise to biases, particularly in pain evaluation. Nevertheless, this is the first study to describe the results of an arthroscopic glenohumeral suspensioplasty by tenodesis of the biceps in the context of painful GHS in hemiplegics. We believe that this technique, developed for hemiplegic shoulders, could find other indications, such as painful peripheral neurological paralysis of the shoulder, or in the case of multidirectional shoulder instability.

## Conclusion

Our study reports the midterm clinical results of 5 hemiplegic patients who underwent arthroscopic humerus suspensioplasty surgery via biceps tenodesis for painful GHS. The patient satisfaction rate was 80%, pain was reduced at the last follow-up, and the radiographic reduction was maintained over time. These results suggest that this surgical technique may be a therapeutic option for hemiplegic patients with painful HSG.

## Disclaimers:

Funding: No funding was disclosed by the authors.

Conflicts of interest: The authors, their immediate families, and any research foundation with which they are affiliated have not received any financial payments or other benefits from any commercial entity related to the subject of this article.
